# The Pan-American Medical Congress in the United States of Colombia

**Published:** 1892-02

**Authors:** Charles A. L. Reed

**Affiliations:** Secretary-General; Cincinnati


CHARLES A. L. REED, *Secretary-General.*

CINCINNATI, January 15, 1892.

---

### THE PAN-AMERICAN MEDICAL CONGRESS IN THE UNITED STATES OF COLOMBIA.

PURSUANT to nominations by Dr. Pedro M. Ibañez, of Bogota, member of the International Executive Committee for the United States of Colombia, the following organization of the Pan-American Medical Congress has been effected in that country : *Vice President*, Dr. Pio Rengifo, New York ; *Secretaries of Sections—General Medicine*—Dr. Ignacio Guterres Ponce, Paris ; *General Surgery*, Dr. Rafael Rocha Castilla, Bogota ; *Military Medicine and Surgery*, Dr. Abraham Aparicio, Bogota ; *Obstetrics*, Dr. Joaquin Maldanado, Bogota ; *Gynecology and Abdominal Surgery*, Dr. José M. Buendia, Bogota ; *Therapeutics*, Dr. Manuel Plata Azuero, Guaduas ; *Anatomy*, Dr. Joan D. Herrara, Bogota ; *Physiology*, Dr. Antonio Bargas Vega, Bogota ; *Pathology*, Dr. Nicolas Osorio, Bogota ; *Diseases of Children*, Dr. Ant. Gomez Calvo, Bogota ; *Ophthalmology*, Dr. Proto Gomez, Bogota ; *Laryngology and Rhinology*, Dr. Luis Fonnegra, Bogota ; *Otology*, Dr. Carlos

Esguerra, Bogota ; *Dermatology*, Dr. Daniel E. Coronado, Bogota ; *Orthopedics*, Dr. Juan E. Manrique, Bogota ; *Naval Hygiene and Quarantine*, Dr. Gabriel I. Castaneda, Bogota; *General Hygiene and Demogrophy* ——— ; *Mental and Nervous Diseases*, Dr. Pablo Garcia Medina, Bogota ; *Oral and Dental Surgery*, Dr. Guillermo Vargas Paredes, Bogota ; *Medical Pedagogics*, Dr. Jorge Vargas, Bogota ; *Medical Jurisprudence*, Dr. Leoncio Barret, Bogota.

*Auxiliary Committee* (each member being the official representative of the Congress in his respective city): Dr. Nicolás Osorio, Dr. Andris Posada Arango, Dr. Jorge E. Delgado, Dr. Eugenio de la Hoz, Dr. Domingo Cagiao, Dr. José Manuel Rodriguez, Dr. Paulo Emelio Villar, Dr. Felix M. Herández, Dr. Rafael Calvo, Dr. N. Ribôn, Dr. Milceades Castro, Dr. Cayetano Lombana, Dr. José M. Martinez, Dr. Isaias Saavedra, Dr. Severo Forres, Dr. N. Villa, Dr. Everisso Garcia, Dr. Miguel Caicedo, Dr. Emilio Villamizar.

The following medical societies have been elected as auxiliaries of the Congress, *viz.: Academia nacional de Medicina, Academia de Medicina de Medellin, Sociedad de Medicina del Cauca.*

The following journals have been designated as official organs of the Congress, *viz.: Revista Medica, Bogota ; Revista de Higiene, Bogota ; El Agricutor, Bogota ; Boletin de Medicina del Cauca, Cali ; Anales de la Academia de Medicin de Medellin, Medellin.*

The expressed wish of the profession of the United States of Colombia is for a date of meeting during the Colombian Exposition.

CHARLES A. L. REED,
*Secretary-General.*

CINCINNATI, January 17, 1892.

